# The impact of a PERMA model-based positive psychology intervention on fear of stroke recurrence: a randomized controlled trial

**DOI:** 10.3389/fpsyg.2025.1498078

**Published:** 2025-02-26

**Authors:** Yanfang Luo, Zhenzhen Su, Lingyun Zhu, Yujuan Huang, Zhimin Liu, Wangmo Dechen, Bo Xu, Xinyu Gao, Yuping Chen, Yuyu Qiu, Jianru Hao

**Affiliations:** ^1^Department of Neurology, Affiliated Hospital of Jiangnan University, Wuxi, China; ^2^Wuxi School of Medicine, Jiangnan University, Wuxi, China; ^3^School of Nursing, Inner Mongolia University for Nationalities, Tongliao, China; ^4^Department of Basic Medicine, Jiangsu Vocational College of Medicine, Yancheng, China

**Keywords:** stroke, PERMA model, fear of recurrence, positive psychological, positive psychological capital, subjective well-being, quality of life

## Abstract

**Introduction:**

This study aimed to examine the effects of a positive psychological intervention, grounded in the PERMA model, on fear levels, psychological capital, overall well-being, and quality of life among stroke patients.

**Methods:**

A single-blind, two-arm randomized controlled trial with a repeated measures design was conducted at the Affiliated Hospital of Jiangnan University from January to December 2023. A total of 125 patients experiencing fear of stroke recurrence were randomly assigned to either the intervention group (*n* = 63), which received a positive psychological intervention based on the PERMA model, or the control group (*n* = 62), which received standard care. We assessed fear levels, psychological capital, well-being, and quality of life at baseline (T0), on the day of discharge (T1), 2 weeks post-discharge (T2), and 4 weeks post-discharge (T3). The scores of the two groups were compared post-intervention using the Generalized Estimation Equation (GEE) model to analyze the effects of time, group membership, and their interaction.

**Results:**

The intervention group showed statistically significant improvements compared to the control group, including reduced fear levels (T2: *t* = −2.094, *p* = 0.038; T3: *t* = −2.207, *p* = 0.029), increased psychological capital (T2: *t* = 2.053, *p* = 0.042; T3: *t* = 2.820, *p* = 0.006), enhanced well-being (T2: *t* = 2.037, *p* = 0.044; T3: *t* = 2.761, *p* = 0.007), and better quality of life (T2: *t* = 2.083, *p* = 0.039; T3: *t* = 2.453, *p* = 0.016) at both T2 and T3. Additionally, significant time-related changes were observed in fear levels, psychological capital, well-being, and quality of life (χ2 = 45.275, *p* < 0.001; χ2 = 37.848, *p* < 0.001; χ2 = 48.255, *p* < 0.001; χ2 = 34.231, *p* < 0.001, respectively). Notably, the interaction effects were statistically significant for well-being (*p* < 0.05).

**Discussion:**

The PERMA-based intervention had a short-term positive effect, reducing fear levels while enhancing psychological capital, well-being, and quality of life among stroke patients.

**Clinical Trial Registration:**

https://www.chictr.org.cn/showproj.html?proj=230313.

## 1 Introduction

Stroke, also referred to as cerebrovascular accident, is a sudden-onset clinical syndrome characterized by localized brain dysfunction resulting from cerebrovascular lesions (Hankey, 2014), with a notably high incidence in the population (Gan et al., 2017). Individuals who experience a stroke are at significant risk of developing various sequelae, including motor dysfunction (Wang et al., 2020), language impairment (Kpadonou et al., 2013), cognitive deficits (Kaur and Sharma, 2022), and psychological disorders (Guo et al., 2024). These sequelae substantially diminish the quality of life for affected patients and contribute to a considerable disease burden. Stroke is marked by a high prevalence, significant disability, elevated mortality, frequent recurrence, and substantial economic burden (GBD 2019 Stroke Collaborators, 2021). Approximately 17.7% of stroke survivors experience a recurrence within 1 year, and this figure rises to over 30% within 5 years (Hao et al., 2024). Recurrent strokes are associated with higher mortality and disability rates compared to initial strokes, necessitating lifelong rehabilitation and support for most affected individuals. This increased need for care imposes a considerable burden on family life and exerts significant psychological pressure on patients.

Many patients are concerned about the adverse consequences of recurrent strokes, leading to a fear of recurrence. This fear of recurrence pertains to the anxiety surrounding the potential return or progression of existing diseases. It is a typical psychological response to emergency events; however, excessive fear of recurrence can be detrimental to patients (Lee-Jones et al., 1997). Research involving breast cancer survivors has demonstrated that fear of recurrence is frequently correlated with symptoms of depression and anxiety. Prolonged fear of recurrence can result in sustained emotional distress among patients and elevate the risk of developing depression and anxiety (Simard et al., 2013). A binary study involving cancer patients revealed that the effect of fear of recurrence on quality of life (QOL) was substantial, surpassing even the impact of anxiety on QOL (Kim et al., 2012). Consequently, for stroke survivors, fear of recurrence is emerging as a critical clinical and psychological issue that necessitates thorough and focused attention.

Research indicates that the fear of cancer recurrence among patients can be mitigated through positive psychological interventions, consequently enhancing their overall mental health and quality of life (Tauber et al., 2019). Positive psychology focuses on identifying and fostering patients’ positive psychological attributes, mobilizing positive emotions, and modulating their sense of well-being (Kubzansky et al., 2018). However, in the context of stroke survivors, existing research on fear of recurrence is predominantly confined to the diagnosis and treatment of mental illnesses, with a notable absence of systematic positive psychological interventions. Therefore, the scope of research should be expanded to address psychological issues comprehensively and facilitate the transition from traditional psychology to the field of psychological medicine.

The PERMA model, which encompasses positive affect (P), engagement (E), relationships (R), meaning (M), and achievement (A)—collectively referred to as happiness PERMA—represents a significant research development within the field of positive psychology (Donaldson et al., 2021; Lorenz et al., 2023; Grosvenor et al., 2023). The self-management training endorsed by this model is generally more readily accepted by patients compared to conventional symptom-centered treatment approaches (Morris, 2011). Furthermore, this model offers ongoing psychological intervention for patients, identifies latent positive emotions, assists patients in confronting their illness, facilitates the correction of erroneous cognitions, thereby enhancing their quality of life and alleviating negative emotions. Currently, positive psychological interventions grounded in the PERMA model have demonstrated significant clinical efficacy in mitigating the fear of recurrence among patients with AIDS (Luo et al., 2022) and breast cancer (Fang et al., 2023). However, there is a paucity of research examining the applicability of this intervention model in addressing the fear of recurrence among stroke survivors. Consequently, this study aims to evaluate the impact of PERMA model-based positive psychological interventions on the fear of recurrence in stroke patients.

## 2 Materials and methods

### 2.1 Study design

This single-blind, randomized controlled trial was conducted over a duration of 3 months. The study protocol received approval from the Research Ethics Committee of the Affiliated Hospital of Jiangnan University (approval number: LS2023064) and was registered with the Chinese Clinical Trial Registry (ChiCTR2400085278). Written informed consent was obtained from all participants prior to their inclusion in the study. Following the collection of baseline measurements, participants were randomly allocated to either the intervention group (IG) or the control group (CG) using a random number table.

### 2.2 Settings and participants

**Figure 1:**
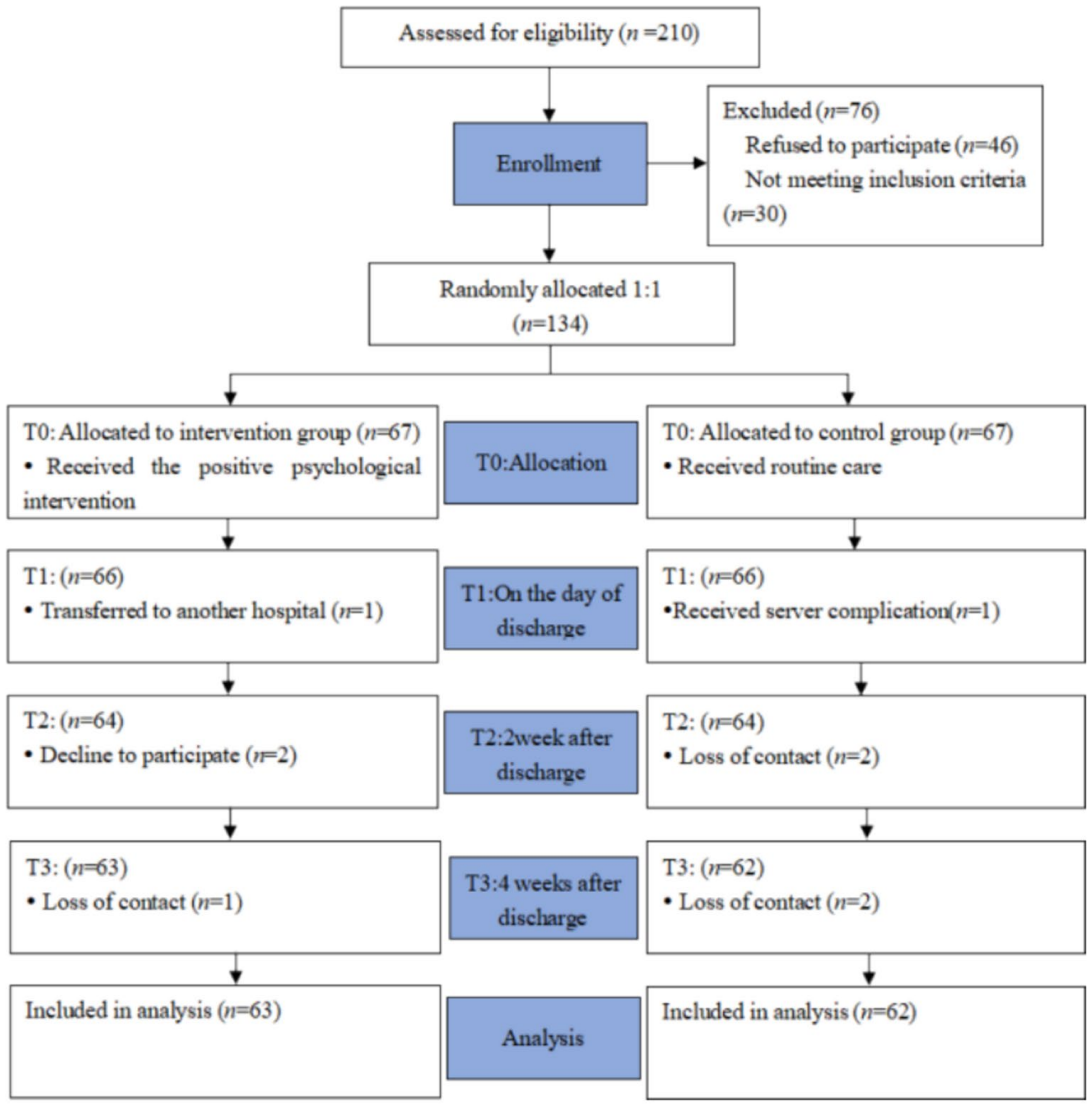
Flow chart of participants’ enrollment.

The study population comprised 125 patients experiencing fear of stroke recurrence, recruited from a tertiary hospital in Wuxi city (Figure 1). The inclusion criteria were as follows: (1) patients who met the diagnostic criteria for acute ischemic stroke, confirmed by head CT or MRI; (2) age ≥ 18 years; (3) a duration of illness of 7 days or more; (4) a Fear of Progression Questionnaire-Short Form (FoP-Q-SF) score of ≥34 points, indicating a clinically significant level of fear; (5) proficiency of patients or their caregivers in operating smartphones; and (6) voluntary participation in the study with signed informed consent. The exclusion criteria were: (1) accompanied by disturbance of consciousness or mental illness; (2) severe aphasia or communication disorder; (3) severe heart, lung, liver, kidney diseases, or malignant tumors; (4) legal blindness or severe visual impairment.

### 2.3 Control group

The control group received standard nursing care, which encompassed the following components: (1) education on stroke for patients; (2) medical treatment for stroke patients; (3) rehabilitation guidance for stroke patients; (4) psychological support for stroke patients; and (5) daily life nursing care for stroke patients.

### 2.4 Intervention group

Based on the control group, a multicomponent exercise nursing intervention was implemented. Formation of the Research Team: The intervention was developed and implemented by a research team composed of 12 members, including a graduate advisor, two neurology professors, one mental health expert, one psychologist, two neurology residents, one rehabilitation specialist, two head nurses from the neurology department, two neurology nurses, and one graduate student. The graduate advisor oversaw the overall study design. The psychologist provided unified guidance and training for stroke patients, coordinated with the head nurses, and managed patient health education. The graduate student, along with the nurses, was responsible for the implementation of the nursing intervention, data collection, interviews, and data analysis. All interventionists completed and passed training in positive psychological nursing.

Development of the Intervention Program: The intervention program was formulated based on a review of relevant literature from the past 5 years on the psychology of fear of recurrence and psychological interventions using the PERMA model for stroke patients. The review aimed to understand the levels of fear, hope, and happiness among stroke patients and assess the effects of these interventions. Based on the literature, an initial positive psychological intervention program was developed using the PERMA model. This program was refined through two rounds of expert consultations conducted via email or face-to-face meetings, and adjustments were made according to expert feedback.

Implementation of the Intervention: The intervention was structured around the five themes of the PERMA model and delivered through seven staged sessions:

1. First Session (Within 3 Days of Admission): The theme was “Positive Emotion.” A 30–45 min one-on-one session was conducted at the bedside using PowerPoint and video presentations. The session focused on addressing psychological issues such as fear of recurrence and emphasizing the importance of cultivating positive emotions.
2. Second Session (Within 4–5 Days of Admission): The theme was “Engaging Happiness.” This 30–45 min session aimed to help patients experience a “flow state” to divert attention from stress and pain through immersive rehabilitation exercises.
3. Third Session (Days 6–7 of Hospitalization): The theme was “Establishing and Maintaining Positive Relationships.” A 30–45 min one-on-one session aimed to teach communication skills, highlight the benefits of positive interactions, and establish communication channels with family members.
4. Fourth Session (Day of Discharge): The theme was “Actively Understanding the Meaning of Life.” This 30–45 min session focused on affirming patients’ self-worth, encouraging active participation in life, and collaboratively planning post-discharge rehabilitation exercises.
5. Fifth Session (Within a Week of Discharge): The theme was “Understanding the Meaning of Life.” Delivered via a 10–15 min online video, this session aimed to maintain patient engagement in their rehabilitation program and provide timely feedback for necessary adjustments.
6. Sixth Session (Within 2 Weeks of Discharge): The theme remained “Understanding the Meaning of Life.” This 10–15 min online video session reinforced the importance of maintaining a structured rehabilitation program and highlighted the positive aspects of patient progress.
7. Seventh Session (Within 3 Weeks of Discharge): The theme was again “Understanding the Meaning of Life.” This 10–15 min online video session aimed to ensure patients actively followed their rehabilitation plans. Patients were encouraged to share daily life videos in a WeChat group to foster a sense of community and positive reinforcement.

Throughout the intervention period, medical staff provided supervision and guidance via WeChat, telephone, or monthly face-to-face consultations. See Table 1 for details.

**Table 1:** Positive psychological intervention program of PERMA model.

Time	Topic	Intervention goals	Content of the intervention
Stage 1: (Admission 1–3 days)	Inject positivity	Establish a trusting relationship. Assess psychological issues related to fear of recurrence. Explain the positive impact of communication.	Conduct the following activities at the patient’s bedside (30–45 min): (1) Address admission-related inquiries and foster a closer relationship with the patient. (2) Evaluate the patient’s understanding of their condition and address any questions. (3) Assess the patient’s physical and mental health status, explain the benefits of positive emotions, and assist with emotional management. (4) Apply the PERMA theory to help patients cultivate positive emotions by encouraging them to articulate affirming phrases, such as: a. Firm belief: Maintain strong conviction; believe you will overcome the illness. b. Face it positively: Confront challenges with bravery, and you will grow stronger. c. Tenacious perseverance: Regardless of the challenges, you can prevail. d. Strengthen your will: With steadfast determination, you will undoubtedly restore your health.
Stage 2: (Admission 4–5 days)	Get into a state of blissful flow	Use the “flow” state to divert attention and relieve stress and pain. Engage in immersive rehabilitation exercises.	Conducted in the ward activity room (30–45 min): Induce the “flow of happiness” through personalized activities. Assess the patient’s needs and hobbies, encouraging the sharing of thoughts and feelings. Engage in the experience of the flow state. Integrate rehabilitation exercises such as bridge exercises, finger stretching, language and swallowing function training. Carry out functional exercises at least twice daily according to the exercise plan.
Stage 3: (Admission 6–7 days)	Build and maintain positive relationships	Understand the benefits of active communication. (2) Learn communication skills.	Conducted in the ward activity room (30–45 min): Assess the patient’s current interpersonal communication status and teach communication skills. The medical team empathizes with the patient. Scenario simulation: train communication skills to deal with stressful situations.
Stage 4: (On the Day of Discharge)	Actively understand the meaning of life	Affirm self-worth and encourage active engagement in life. Develop a post-discharge rehabilitation plan.	Conducted at the patient’s bedside (30–45 min): Actively explain the meaning of life, jointly taught by psychotherapists and doctors. Distribute discharge education brochures, encouraging patients to share their happiness with others. Develop a personalized discharge exercise plan.
Stage 5: (2 Weeks After Discharge)	Get a sense of accomplishment	Actively follow the rehabilitation plan. Pursue set life goals. Provide timely feedback and adjust the rehabilitation plan as needed.	Telephone follow-up (10–15 min): Encourage patients to maintain good living habits. Encourage family members to provide a supportive family environment. Guide patients to set realistic rehabilitation goals and share their progress. Encourage family members to record videos of patients participating in daily household chores such as washing dishes or sweeping the floor.
Stage 6: (3 Weeks After Discharge)		Follow the rehabilitation plan confidently. Complete set goals. Provide timely feedback to adjust the rehabilitation plan.	Telephone follow-up (10–15 min): Instruct patients to conduct self-training according to the rehabilitation plan. Encourage patients to complete a favorite activity every day or every 2 days, such as calligraphy, cooking, or reading.
Stage 7: (4 Weeks After Discharge)		Maintain confidence while following the rehabilitation plan. Complete goals. Provide positive reinforcement to others.	Telephone follow-up (10–15 min): Provide patients with affirmation and support for their rehabilitation efforts. Encourage patients to continue engaging in activities they excel at and experience a sense of accomplishment. Guide patients to record and share daily life moments.

### 2.5 Data collection and outcomes measures

This study utilized on-site surveys, with researchers trained in standardized instructional language for participant engagement. The study objectives and key points were clearly communicated to the researchers. Stroke patients independently and anonymously completed questionnaires during on-site visits, which were subsequently subjected to quality control by a designated officer. During the online follow-up intervention, follow-up nurses collected weekly data on the frequency and duration of patients’ online logins.

Data were collected at three time points: baseline, week 4, and week 8. The Fear of Disease Progression to Simplify Scale (Fear of Progression Questionnaire - Short Form, FoP - Q - SF) was established in 2006 by German scholars, such as Mehnert, on the basis of the FoP – Q (Mehnert et al., 2006). The simplified scale includes two dimensions: physical health and social family function. Each dimension has six items, for a total of 12 items. According to the Likert 5-level scoring method, each item is assigned a score of 1–5, ranging from “strongly disagree” to “strongly agree.” The total score ranges from 12 to 60. A higher score indicates a higher level of fear in the patient. A total score ≥ 34 indicates that patients have a fear of recurrence after stroke. The Cronbach’s *α* coefficient for the scale exceeded 0.88.

The Positive Psychological Capital Questionnaire (PPQ) was developed by Zhang. It comprises four dimensions (self-efficacy, hope, resilience, and optimism) and a total of 36 items (Sood and Puri, 2023). Ten questions assess population and sociological factors, while 26 questions assess the four dimensions. Each factor is scored on a seven-level scale ranging from “completely inconsistent” to “completely consistent.” Higher scores indicate higher levels of positive psychological capital. The Cronbach’s *α* coefficient for the scale exceeds 0.92.

The subjective well-being scale used to measure patients’ subjective well-being was developed by Campbell (1976) in 1976 and translated into Chinese by Fan Xiaodong. It primarily assesses the patient’s subjective well-being, including the general affective index scale (8 items) and the general life satisfaction questionnaire (1 item). Low well-being scores range from 2.1 to 6, while scores from 6.1 to 10 indicate moderate well-being, and scores from 10.1 to 14.7 indicate high well-being. Higher scores on the scale signify greater well-being, and the Cronbach’s *α* coefficient of the scale is 0.90.

The Stroke Specific Quality of Life Scale (SS-QoL) was employed to evaluate quality of life (QoL) (Muus et al., 2007). The SS-QoL is a specific instrument designed to assess health-related QoL in individuals who have experienced a stroke. The scale consists of two dimensions: physical health-related quality of life and psychosocial health-related quality of life, with a total of 12 items. A 5-point Likert scale is used, where the physical health-related quality of life dimension is scored from 1 (not at all) to 5 (no difficulty), and the psychosocial health-related quality of life dimension is scored from 1 (strongly agree) to 5 (strongly disagree). The total score ranges from 12 to 60, with higher scores indicating higher levels of QoL. The Cronbach’s α coefficient of the scale is 0.850.

### 2.6 Statistical analysis

The data underwent analysis using SPSS version 26.0 (IBM SPSS Data Collection, New York, NY, USA). Statistical analysis involved the calculation of mean, standard deviation, frequency, and percentage. Following an assessment of normality for the variables, independent t-tests, chi-square tests, or Fisher’s exact tests were applied to compare normally distributed variables between the two groups, as appropriate. The overall comparison of outcome indicators between the intervention and control groups was evaluated using the Generalized Estimation Equation Model (GEE). Differences and trends in various indicators among patients in both groups were analyzed, focusing on the main effects of the group, time, and their interaction. The analysis employed the linearization method for generalized estimation equations, utilized an unstructured working correlation matrix, and applied the least significant difference method for posttest comparisons. This research utilized a two-sided test, establishing statistical significance at a *p*-value of less than 0.05.

### 2.7 Sample size

The calculation of the sample size utilized the two-sample mean formula: n_1_ = n_2_ = 2[(μ_α_ + μ_β_)*σ*/*δ*]^2^. Each primary indicator (including fear level, positive psychological capital, sense of well-being, and quality of life) was individually assessed to determine the necessary sample size. The largest required sample size, derived from fear level, was established as the sample capacity for this research. Based on findings from a previous similar study (σ = 6.41, δ = 3.96) (Chen et al., 2024), the minimum number of participants needed was 55 per group. Additionally, accounting for a 10% attrition rate, a total of at least 122 patients were required, with 61 participants in each group.

## 3 Results

A total of 210 participants were screened prior to the study, of which 134 volunteers who met the research criteria were randomly assigned to either the intervention group (*n* = 67) or the control group (*n* = 67). Of these, 125 participants (93.28%) successfully completed the three-week follow-up data collection, comprising 63 individuals in the intervention group and 62 in the control group. Nine participants (6.72%) withdrew from the study. The reasons for attrition included transfer to another hospital (Hankey, 2014), serious postoperative complications (Hankey, 2014), loss of contact (Kaur and Sharma, 2022), and refusal to participate in the investigation (Gan et al., 2017).

Table 2 provides a comprehensive overview of the baseline sociodemographic characteristics of the participants (*n* = 125). The mean age of the patients was 66.66 ± 12.92 years, with a range from 30 to 88 years. More than half of the patients were male (53.60%), and a significant majority resided in urban areas (66.40%). A large proportion of patients had attained a junior high school education level or higher (79.20%). The majority of participants were married (83.20%), and over a quarter were employed (29.60%). Medical insurance for urban employees or residents was the most prevalent form of health insurance (61.60%). Approximately 71.20% of patients reported a monthly household income of 3,000 RMB or higher per person. Furthermore, the vast majority of participants were not addicted to tobacco or alcohol (89.60 and 93.60%, respectively). Regarding chronic disease prevalence, over half of the patients had hypertension (61.60%), while more than a quarter were affected by diabetes (32.00%). Before intervention, baseline comparisons between the two patient groups revealed no significant differences in their characteristics.

**Table 2:** Demographic and clinical traits of the participants.

Variables	Total (*n* = 125)	Intervention group (*n* = 63)	Control group (*n* = 62)	*t*/*χ^2^*/*Z*- value	*P-*value
Age (years, mean ± SD)	66.66 ± 12.92	66.67 ± 13.74	66.65 ± 12.14	0.009 ^a^	0.993
Gender (*n*, %)				0.007 ^b^	0.934
Male	67 (53.60)	34 (53.97)	33 (53.23)		
Female	58 (46.40)	29 (46.03)	29 (46.77)		
Educational level (*n*, %)				0.632 ^b^	0.889
Primary and below	26 (20.80)	12 (19.05)	14 (22.58)		
Junior high school	65 (52.00)	33 (52.38)	32 (51.61)		
High school	25 (20.00)	14 (22.22)	11 (17.74)		
College and above	9 (7.20)	4 (6.35)	5 (8.06)		
Marital status (*n*,%)				0.459 ^b^	0.498
Married	104 (83.20)	51 (80.95)	53 (85.48)		
Unmarried/Widowed	21 (16.80)	12 (19.05)	9 (14.52)		
Residence (*n*,%)				2.106 ^b^	0.147
Urban	83 (66.40)	38 (60.32)	45 (72.58)		
Countryside	42 (33.60)	25 (39.68)	17 (27.42)		
Work status (*n*,%)				2.052 ^b^	0.358
On job	37 (29.60)	16 (25.40)	21 (33.87)		
Retired	62 (49.60)	31 (49.21)	31 (50.00)		
Unemployed	26 (20.80)	16 (25.40)	10 (16.13)		
Health insurance (*n*, %)				0.614 ^b^	0.736
New rural cooperative medical insurance	10 (8.00)	6 (9.52)	4 (6.45)		
Medical insurance for urban employees/residents	77 (61.60)	37 (58.73)	40 (64.52)		
Other payment methods	38 (30.40)	20 (31.75)	18 (29.03)		
Monthly income per person (RMB) (*n*, %)				2.499 ^b^	0.287
<3,000	36 (28.80)	19 (30.16)	17 (27.42)		
3,000–5,000	62 (49.60)	34 (53.97)	28 (45.16)		
≥5,000	27 (21.60)	10 (15.87)	17 (27.42)		
Smoking (*n*, %)				0.105 ^b^	0.746
No	112 (89.60)	57 (90.48)	55 (88.71)		
Yes	13 (10.40)	6 (9.52)	7 (11.29)		
Alcohol drinking (*n*, %)				0.574 ^c^	0.491
No	117 (93.60)	60 (95.24)	57 (91.94)		
Yes	8 (6.40)	3 (4.76)	5 (8.06)		
Body mass index (BMI) (kg/m^2^, mean ± SD)	24.66 ± 3.04	24.84 ± 3.14	24.49 ± 2.95	0.642 ^a^	0.522
Hypertension (*n*, %)				0.192 ^b^	0.661
No	48 (38.40)	23 (36.51)	25 (40.32)		
Yes	77 (61.60)	40 (63.49)	37 (59.68)		
Diabetes (*n*, %)				0.686 ^b^	0.407
No	85 (68.00)	45 (71.43)	40 (64.52)		
Yes	40 (32.00)	18 (28.57)	22 (35.48)		
Heart disease (*n*, %)				0.821 ^c^	0.396
No	112 (89.60)	58 (92.06)	54 (87.10)		
Yes	13 (10.40)	5 (7.94)	8 (12.90)		

Other forms of payment included public and self-funding. SD, standard deviation; IQR, interquartile range. ^a^ Independent samples *t*-test. ^b^ Chi-squared test. ^c^ Fisher exact test.

**Figure 2:**
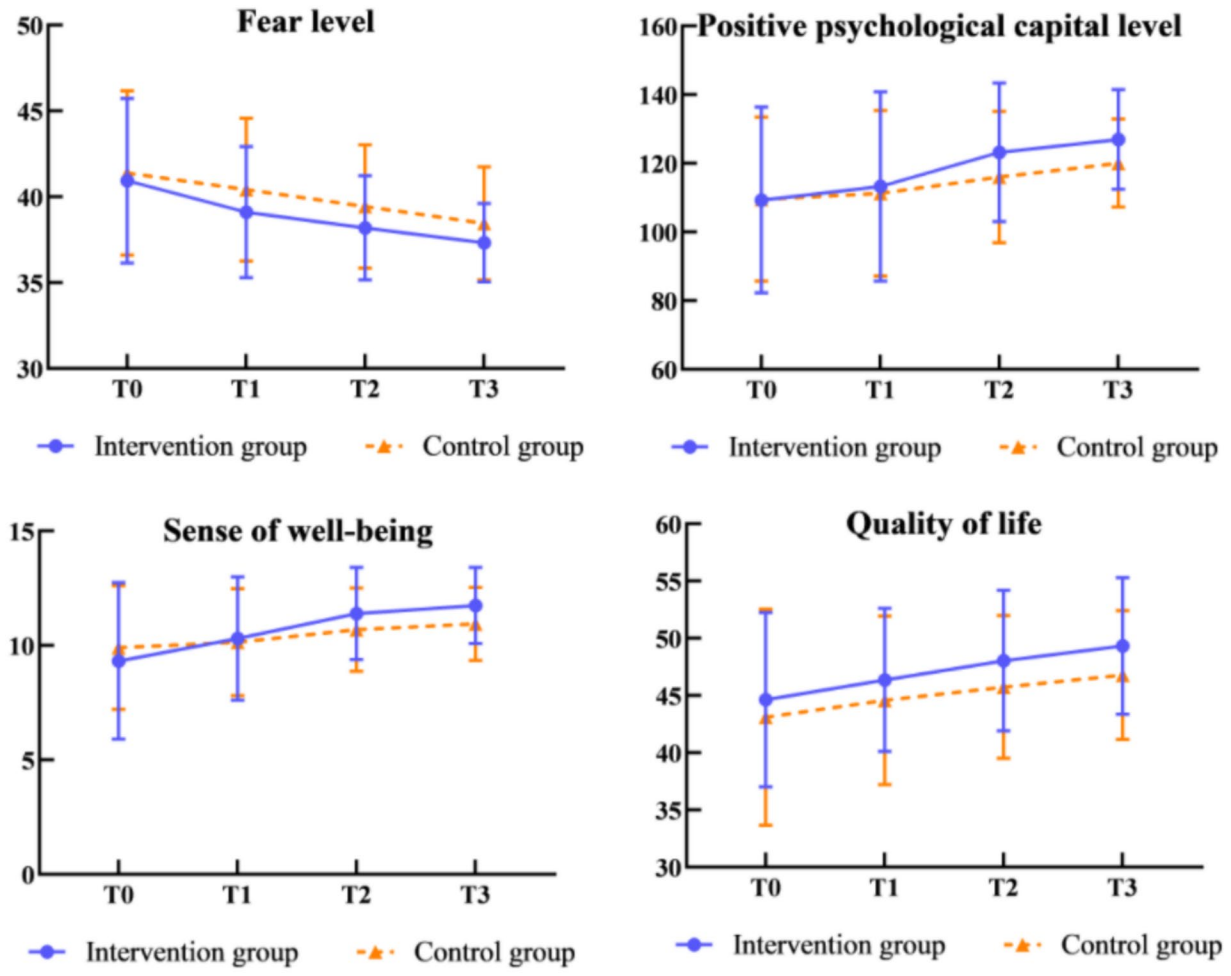
Change in mean scores of patients’ fear level, positive psychological capital level, sense of well-being and quality of life.

Tables 3, 4 present the outcomes of patients at baseline and the three follow-up assessments. Figure 2 illustrates the changes in the mean and standard deviation of scores for fear level, positive psychological capital, sense of well-being, and quality of life over time. At baseline, no statistically significant differences were observed between the intervention and control groups in terms of fear level (*t* = −0.526, *p* = 0.600), positive psychological capital (*t* = −0.047, *p* = 0.963), sense of well-being (*t* = −1.057, *p* = 0.293), and quality of life (*t* = 1.004, *p* = 0.317). The Generalized Estimating Equations (GEE) analysis further confirmed that there were no statistically significant differences between the two groups regarding fear level (χ^2^ = 3.188, *p* = 0.077), positive psychological capital (χ^2^ = 1.338, *p* = 0.250), sense of well-being (χ^2^ = 0.524, *p* = 0.470), and quality of life (χ^2^ = 3.339, *p* = 0.070).

**Table 3:** Testing the impacts of Generalized Estimation Equation (GEE) Models on patients’ fear level, positive psychological capital level, sense of well-being and quality of life.

Variables	Effect	Wald *χ^2^*	*P-*value
Fear level	Group	3.188	0.077
	Time	45.275	<0.001
	Group _*_ Time	0.903	0.409
Positive psychological capital level	Group	1.338	0.250
	Time	37.848	<0.001
	Group _*_ Time	3.029	0.057
Sense of well-being	Group	0.524	0.470
	Time	48.255	<0.001
	Group _*_ Time	7.808	0.001
Quality of life	Group	3.339	0.070
	Time	34.231	<0.001
	Group _*_ Time	0.563	0.538

**Table 4:** Comparison of fear level, positive psychological capital level, sense of well-being and quality of life between two groups of patients.

Variables	Time	Intervention group (mean ± SD)	Control group (mean ± SD)	*t*-value	*P-*value
Fear level	T0	40.94 ± 4.79	41.39 ± 4.79	−0.526	0.600
	T1	39.11 ± 3.81	40.42 ± 4.16	−1.833	0.069
	T2	38.19 ± 3.03	39.44 ± 3.59	−2.094	0.038
	T3	37.33 ± 2.28	38.45 ± 3.29	−2.207	0.029
Positive psychological capital level	T0	109.33 ± 27.04	109.55 ± 23.88	−0.047	0.963
	T1	113.27 ± 27.56	111.26 ± 24.08	0.434	0.665
	T2	123.21 ± 20.13	116.00 ± 19.10	2.053	0.042
	T3	126.97 ± 14.52	120.06 ± 12.78	2.820	0.006
Sense of well-being	T0	9.32 ± 3.42	9.90 ± 2.70	−1.057	0.293
	T1	10.30 ± 2.69	10.14 ± 2.34	0.355	0.723
	T2	11.39 ± 2.01	10.69 ± 1.82	2.037	0.044
	T3	11.74 ± 1.66	10.94 ± 1.59	2.761	0.007
Quality of life	T0	44.63 ± 7.61	43.10 ± 9.43	1.004	0.317
	T1	46.35 ± 6.25	44.58 ± 7.36	1.450	0.150
	T2	48.05 ± 6.13	45.74 ± 6.24	2.083	0.039
	T3	49.33 ± 5.97	46.79 ± 5.62	2.453	0.016

Meanwhile, comparisons between the groups indicated that patients in the intervention group exhibited lower fear levels (T2: *t* = −2.094, *p* = 0.038; T3: *t* = −2.207, *p* = 0.029), higher levels of positive psychological capital (T2: *t* = 2.053, *p* = 0.042; T3: *t* = 2.820, *p* = 0.006), enhanced sense of well-being (T2: *t* = 2.037, *p* = 0.044; T3: *t* = 2.761, *p* = 0.007), and improved quality of life (T2: *t* = 2.083, *p* = 0.039; T3: *t* = 2.453, *p* = 0.016) at T2 and T3. Notably, fear level, positive psychological capital, sense of well-being, and quality of life exhibited significant changes over time (χ^2^ = 45.275, *p* < 0.001; χ^2^ = 37.848, *p* < 0.001; χ^2^ = 48.255, *p* < 0.001; and χ^2^ = 34.231, *p* < 0.001, respectively). The interaction effects were statistically significant for sense of well-being (*p* < 0.05).

## 4 Discussion

Patients who have experienced a stroke must contend not only with the abrupt onset, uncertainty, and potential life-threatening nature of recurrent cerebrovascular events but also with the subsequent risks of additional physical function loss, language impairment, cognitive deficits, and other sequelae. Furthermore, they face numerous challenges and difficulties during the rehabilitation process (Ashley et al., 2019). Compared to other diseases, stroke patients are more prone to experiencing negative emotions such as apathy, a sense of loss, and self-doubt. Individuals may perceive that their lives and futures are dominated by their illness, resulting in a diminished capacity to live as freely as they once did. Prolonged mental fatigue can contribute to anxiety, depression, and other psychological issues, thereby undermining patients’ self-efficacy, diminishing their hope, increasing feelings of vulnerability, and eroding their resilience and optimism. These psychological effects can further impede the rehabilitation process, degrade the quality of life, and reduce overall well-being and happiness (Kusec et al., 2023). To the best of our knowledge, this study represents the first randomized controlled trial to implement a positive psychology program based on the PERMA model in stroke patients. The findings indicate that the program significantly reduces patients’ fear of recurrence, enhances their positive psychological capital, improves subjective well-being, and ultimately enhances their quality of life.

A recent meta-analysis demonstrated that PERMA-based positive psychology interventions can effectively alleviate patients’ fear of relapse, corroborating the findings of this study (Chen et al., 2024). The PERMA framework in positive psychology interventions focuses on eliciting internal positive psychological emotions and fostering the regulation of optimistic emotions over negative ones. This approach aims to enhance patients’ overall life perception and cultivate their positive qualities, thereby effectively mitigating their fear of the disease. During the intervention phase of this study, training focused on fostering close relationships can assist patients in identifying more meaningful goals, engaging in stimulating activities, and recognizing opportunities for personal growth within the context of rehabilitation and family life. This approach can enhance their sense of responsibility toward society, friendships, and family, thereby promoting the development of a more comprehensive and scientifically informed outlook on life and values. Consequently, this may mitigate patients’ preoccupation with the fear of recurrence to some extent (Niu et al., 2017). In the advanced phase of intervention, directing patients to engage in personalized functional exercises and dynamically adjusting their exercise goals can substantially enhance their rehabilitation awareness. This approach encourages patients to focus on their abilities, actively explore their potential, and proactively engage in self-healing practices. Consequently, it effectively reduces patients’ fear of recurrence and improves both their physical and social functioning (Loupis and Faux, 2013; Chockalingam et al., 2022).

The findings of this study indicated that the experimental group exhibited a higher level of positive psychological capital post-intervention compared to the control group, aligning with the outcomes of previous research (Carod-Artal and Egido, 2009; Buijck et al., 2014). This effect may be attributed to the positive psychological intervention grounded in the PERMA model, which emphasizes patients’ internal experiences and aims to enhance their positive cognitive appraisal of the disease. The intervention team conducted a series of structured positive belief correction interviews to address patients’ psychological distress at various stages. Additionally, they facilitated opportunities for patients to engage in active communication with peers and family members, thereby promoting the enhancement of interpersonal relationships, self-esteem, and confidence. This comprehensive approach aimed to progressively rectify patients’ misconceptions and negative cognitions related to their illness. Through face-to-face interviews and written recordings, patients were encouraged to express themselves, share their energy with others, and engage in stimulating activities such as singing aloud, playing chess, and other positive events. These activities were designed to foster the development of a positive mindset (Kovich et al., 2023). By enhancing beliefs and confidence, this approach aims to reduce symptoms of depression and improve overall mood, thereby ultimately enhancing the patient’s positive psychological capital.

In recent years, the comprehensive application of positive psychology research has highlighted the significance of well-being as a critical metric for assessing the psychological state of individuals post-treatment. The current study demonstrates that positive psychological interventions grounded in the PERMA model effectively enhance patient well-being, corroborating findings from previous research. The PERMA model provides a comprehensive definition of happiness and delineates five distinct components of well-being: positive emotions, engagement, relationships, meaning, and achievement. These measurable factors collectively contribute to enhancing an individual’s well-being and can effectively mitigate negative thinking, particularly in challenging circumstances (Weiss et al., 2024). Furthermore, researchers persist in imparting hope for a cure to patients, while providing positive incentives and support for personal attributes. This approach not only effectively rectifies patients’ perceptions of negative emotions but also diminishes the self-perceived burden associated with the disease. Consequently, it alleviates fear and anxiety related to the illness, enhances self-esteem, improves self-management capabilities, and ultimately augments overall well-being (Oshimi et al., 2023).

The residual dysfunction following a stroke, coupled with the apprehension of recurrence, significantly impacts patients’ quality of life, which is a crucial indicator of individual well-being. Previous research has explored the effects of a positive psychological intervention grounded in the PERMA model on breast cancer patients. The findings affirm that this program can enhance patients’ quality of life and demonstrates promising potential for broader application (Fang et al., 2023). The findings of this study indicated that post-intervention scores in the physical health dimension, social and family dimension, and the overall FoP-Q-SF were significantly lower in the study group compared to the control group. This outcome may be attributed to the enhancement of motor function recovery and psychological status among patients in the study group, achieved through the implementation of multiple intervention methods. Firstly, engaging in physical exercise can significantly enhance the recovery of physical activity (Manning et al., 2022; Lee et al., 2022; Bahouth et al., 2023), augment patients’ sense of control over their own behavior, and facilitate a transition from passive to active engagement, thereby enabling them to experience the benefits of proactive efforts. Furthermore, the implementation of positive psychological interventions can effectively mobilize the enthusiasm of both patients and their families, fostering increased interaction between them. This enhanced interaction contributes to a notable improvement in the patients’ overall quality of life (Farhadi et al., 2014; Zhang et al., 2023).

Our study is subject to several limitations. Firstly, the duration of the randomized controlled trials was relatively short, necessitating further long-term follow-up to evaluate the enduring effects of the intervention. Secondly, the patient sample was confined to a tertiary hospital in Wuxi, potentially restricting the generalizability of the findings. Future research should incorporate multi-center and large-sample clinical trials to substantiate the feasibility and efficacy of this intervention. Furthermore, the control group in this study was administered standard care as opposed to a targeted psychological intervention. It is advisable that subsequent research endeavors compare positive psychological interventions with alternative interventions to more robustly substantiate the efficacy of the former.

## 5 Conclusion

The findings of this study suggest that the positive psychological intervention grounded in the PERMA model is both effective and feasible. This intervention significantly reduces patients’ fear of recurrence and enhances their positive psychological capital, happiness index, and overall quality of life when compared to routine care.

## Data Availability

The raw data supporting the conclusions of this article will be made available by the authors, without undue reservation.

## References

[ref1] Ashley K. D. Lee L. T. Heaton K. (2019). Return to work among stroke survivors. Workplace Health Saf. 67, 87–94. doi: 10.1177/2165079918812483, PMID: 30616463

[ref2] Bahouth M. N. Deluzio S. Pruski A. Zink E. K. (2023). Nonpharmacological treatments for hospitalized patients with stroke: a nuanced approach to prescribing early activity. Neurotherapeutics 20, 712–720. doi: 10.1007/s13311-023-01392-2, PMID: 37289401 PMC10275818

[ref3] Buijck B. I. Zuidema S. U. Spruit-van Eijk M. Bor H. Gerritsen D. L. Koopmans R. T. (2014). Determinants of geriatric patients' quality of life after stroke rehabilitation. Aging Ment. Health 18, 980–985. doi: 10.1080/13607863.2014.899969 24679003

[ref4] Campbell A. (1976). Subjective measures of well-being. Am. Psychol. 31, 117–124. doi: 10.1037/0003-066X.31.2.117 1267244

[ref5] Carod-Artal F. J. Egido J. A. (2009). Quality of life after stroke: the importance of a good recovery. Cerebrovasc. Dis. 27, 204–214. doi: 10.1159/000200461, PMID: 19342853

[ref6] Chen Z. Luo H. Xu L. Yi Y. (2024). Machine learning model for predicting stroke recurrence in adult stroke patients with moyamoya disease and factors of stroke recurrence. Clin. Neurol. Neurosurg. 242:108308. doi: 10.1016/j.clineuro.2024.108308, PMID: 38733759

[ref7] Chen J. Sun Y. H. Shao Y. Meng A. F. Bai Y. M. Li X. L.. (2024). Effects of psychological interventions on fear of cancer recurrence: a systematic review and network meta-analysis. Precision Med. Sci. 13, 84–98. doi: 10.1002/prm2.12131

[ref8] Chockalingam M. Vasanthan L. T. Balasubramanian S. Sriram V. (2022). Experiences of patients who had a stroke and rehabilitation professionals with upper limb rehabilitation robots: a qualitative systematic review protocol. BMJ Open 12:e065177. doi: 10.1136/bmjopen-2022-065177, PMID: 36123077 PMC9486398

[ref9] Donaldson S. I. van Zyl L. E. Donaldson S. I. (2021). PERMA+4: a framework for work-related wellbeing, performance and positive organizational psychology 2.0. Front. Psychol. 12:817244. doi: 10.3389/fpsyg.2021.817244, PMID: 35140667 PMC8819083

[ref10] Fang H. Zeng Y. Liu Y. Zhu C. (2023). The effect of the PERMA model-based positive psychological intervention on the quality of life of patients with breast cancer. Heliyon 9:e17251. doi: 10.1016/j.heliyon.2023.e17251, PMID: 37416631 PMC10320023

[ref11] Farhadi M. Reisi-Dehkordi N. Kalantari M. Zargham-Boroujeni A. (2014). Efficacy of group meaning centered hope therapy of cancer patients and their families on patients' quality of life. Iran. J. Nurs. Midwifery Res. 19, 290–294. 24949069 PMC4061631

[ref12] Gan Y. Wu J. Zhang S. Li L. Yin X. Gong Y.. (2017). Prevalence and risk factors associated with stroke in middle-aged and older Chinese: a community-based cross-sectional study. Sci. Rep. 7:9501. doi: 10.1038/s41598-017-09849-z, PMID: 28842623 PMC5572736

[ref13] GBD 2019 Stroke Collaborators (2021). Global, regional, and national burden of stroke and its risk factors, 1990-2019: a systematic analysis for the global burden of disease study 2019. Lancet Neurol. 20, 795–820. doi: 10.1016/S1474-4422(21)00252-0, PMID: 34487721 PMC8443449

[ref14] Grosvenor L. P. Errichetti C. L. Holingue C. Beasley J. B. Kalb L. G. (2023). Self-report measurement of well-being in autistic Adults: psychometric properties of the PERMA profiler. Autism Adulthood 5, 401–410. doi: 10.1089/aut.2022.0049, PMID: 38116049 PMC10726181

[ref15] Guo Y. Zhou M. Yan X. Liu Y. Wang L. (2024). Latent class analysis and longitudinal development trajectory study of psychological distress in patients with stroke: a study protocol. Front. Psych. 15:1326988. doi: 10.3389/fpsyt.2024.1326988, PMID: 38887726 PMC11181843

[ref16] Hankey G. J. (2014). Secondary stroke prevention. Lancet Neurol. 13, 178–194. doi: 10.1016/S1474-4422(13)70255-2 24361114

[ref17] Hao J. Qian L. Ye F. Luo Y. Xu C. Wang J.. (2024). Factors influencing physical activity levels in elderly community-dwelling convalescent stroke survivors: a cross-sectional study. Geriatr. Nurs. 58, 472–479. doi: 10.1016/j.gerinurse.2024.06.017, PMID: 38955038

[ref18] Kaur M. Sharma S. (2022). Molecular mechanisms of cognitive impairment associated with stroke. Metab. Brain Dis. 37, 279–287. doi: 10.1007/s11011-022-00901-0 35029798

[ref19] Kim Y. Carver C. S. Spillers R. L. Love-Ghaffari M. Kaw C. K. (2012). Dyadic effects of fear of recurrence on the quality of life of cancer survivors and their caregivers. Qual. Life Res. 21, 517–525. doi: 10.1007/s11136-011-9953-0, PMID: 21691928

[ref20] Kovich M. K. Simpson V. L. Foli K. J. Hass Z. Phillips R. G. (2023). Application of the PERMA model of well-being in undergraduate students. Int. J. Commun. Wellbeing. 6, 1–20. doi: 10.1007/s42413-022-00184-4, PMID: 36320595 PMC9607835

[ref21] Kpadonou G. T. Alagnidé E. Niama-Natta D. Houngbédji G. Adjaka N. (2013). Verbal communication disorders in brain damaged post-stroke patients in Benin. Ann. Phys. Rehabil. Med. 56, 663–672. doi: 10.1016/j.rehab.2013.08.004, PMID: 24210528

[ref22] Kubzansky L. D. Huffman J. C. Boehm J. K. Hernandez R. Kim E. S. Koga H. K.. (2018). Positive psychological well-being and cardiovascular disease: JACC health promotion series. J. Am. Coll. Cardiol. 72, 1382–1396. doi: 10.1016/j.jacc.2018.07.042, PMID: 30213332 PMC6289282

[ref23] Kusec A. Milosevich E. Williams O. A. Chiu E. G. Watson P. Carrick C.. (2023). Long-term psychological outcomes following stroke: the OX-CHRONIC study. BMC Neurol. 23:426. doi: 10.1186/s12883-023-03463-5, PMID: 38036966 PMC10688008

[ref24] Lee K. E. Choi M. Jeoung B. (2022). Effectiveness of rehabilitation exercise in improving physical function of stroke patients: a systematic review. Int. J. Environ. Res. Public Health 19. doi: 10.3390/ijerph191912739, PMID: 36232038 PMC9566624

[ref25] Lee-Jones C. Humphris G. Dixon R. Hatcher M. B. (1997). Fear of Cancer recurrence — a literature review and proposed cognitive formulation to explain exacerbation of recurrence fears. Psychooncology 6, 95–105. doi: 10.1002/(SICI)1099-1611(199706)6:2<95::AID-PON250>3.0.CO;2-B, PMID: 9205967

[ref26] Lorenz T. Ho J. Beyer M. Hagitte L. (2023). Measuring PERMA+4: validation of the German version of the positive functioning at work scale. Front. Psychol. 14:1231299. doi: 10.3389/fpsyg.2023.1231299, PMID: 37637923 PMC10448252

[ref27] Loupis Y. M. Faux S. G. (2013). Family conferences in stroke rehabilitation: a literature review. J. Stroke Cerebrovasc. Dis. 22, 883–893. doi: 10.1016/j.jstrokecerebrovasdis.2012.12.003, PMID: 23352687

[ref28] Luo L. Li Y. Zhou Z. Yang S. Qin Y. Peng H.. (2022). Study on the effect of positive psychological intervention based on PERMA model on perioperative patients with AIDS complicated with breast Cancer. Comput. Math. Methods Med. 2022, 1–8. doi: 10.1155/2022/9788122, PMID: 35979048 PMC9377935

[ref29] Manning K. Jennings S. Pearson M. Sloane R. Hall K. Bourassa K.. (2022). Can virtual exercise promote physical resilience during the covid-19 pandemic among active older adultS? Innov. Aging 6:415. doi: 10.1093/geroni/igac059.1631, PMID: 38779614

[ref30] Mehnert A. Herschbach P. Berg P. Henrich G. Koch U. (2006). Progredienzangst bei Brustkrebspatientinnen - Validierung der Kurzform des Progredienzangstfragebogens PA-F-KF/ fear of progression in breast cancer patients – validation of the short form of the fear of progression questionnaire (FoP-Q-SF). Z. Psychosom. Med. Psychother. 52, 274–288. doi: 10.13109/zptm.2006.52.3.274, PMID: 17156600

[ref31] Morris R. (2011). The psychology of stroke in young adults: the roles of service provision and return to work. Stroke Res Treat. 2011:534812. doi: 10.4061/2011/534812, PMID: 21423559 PMC3056452

[ref32] Muus I. Williams L. S. Ringsberg K. C. (2007). Validation of the stroke specific quality of life scale (SS-QOL): test of reliability and validity of the Danish version (SS-QOL-DK). Clin. Rehabil. 21, 620–627. doi: 10.1177/0269215507075504, PMID: 17702704

[ref33] Niu J. M. Kong F. Z. Zhang Y. T. Shang Y. X. (2017). Study on the correlation between depression and emotion regulation strategies in the elderly residents. Zhonghua Liu Xing Bing Xue Za Zhi 38, 1611–1615. doi: 10.3760/cma.j.issn.0254-6450.2017.12.005, PMID: 29294572

[ref34] Oshimi D. Kinoshita K. Yamashita R. (2023). The mediating role of sport-specific PERMA in the relationship between physical activity/passive sport and global well-being/loneliness. J. Leis. Res. 56, 274–295. doi: 10.1080/00222216.2023.2287009, PMID: 39935898

[ref35] Simard S. Thewes B. Humphris G. Dixon M. Hayden C. Mireskandari S.. (2013). Fear of cancer recurrence in adult cancer survivors: a systematic review of quantitative studies. J. Cancer Surviv. 7, 300–322. doi: 10.1007/s11764-013-0272-z, PMID: 23475398

[ref36] Sood S. Puri D. (2023). Psychological capital and positive mental health of student-athletes: psychometric properties of the sport psychological capital questionnaire. Curr. Psychol. 42, 21759–21774. doi: 10.1007/s12144-022-03272-y

[ref37] Tauber N. M. O'Toole M. S. Dinkel A. Galica J. Humphris G. Lebel S.. (2019). Effect of psychological intervention on fear of Cancer recurrence: a systematic review and Meta-analysis. J. Clin. Oncol. 37, 2899–2915. doi: 10.1200/JCO.19.00572, PMID: 31532725 PMC6823887

[ref38] Wang C. Peng L. Hou Z. G. Li J. Zhang T. Zhao J. (2020). Quantitative assessment of upper-limb motor function for post-stroke rehabilitation based on motor synergy analysis and multi-modality fusion. IEEE Trans. Neural Syst. Rehabil. Eng. 28, 943–952. doi: 10.1109/TNSRE.2020.2978273, PMID: 32149692

[ref39] Weiss E. L. Donaldson S. I. Reece A. (2024). Well-being as a predictor of academic success in student veterans and factor validation of the PERMA + 4 well-being measurement scale. J. Am. Coll. Heal. 1-8, 1–8. doi: 10.1080/07448481.2023.2299417 38227924

[ref40] Zhang Y. Tang R. Bi L. Wang D. Li X. Gu F.. (2023). Effects of family-centered positive psychological intervention on psychological health and quality of life in patients with breast cancer and their caregivers. Support Care Cancer 31:592. doi: 10.1007/s00520-023-08053-2, PMID: 37750931

